# Hypertension Associated With Hyperlipidemia Induced Different MicroRNA Expression Profiles in Plasma, Platelets, and Platelet-Derived Microvesicles; Effects of Endothelial Progenitor Cell Therapy

**DOI:** 10.3389/fmed.2019.00280

**Published:** 2019-12-03

**Authors:** Nicoleta Alexandru, Alina Constantin, Miruna Nemecz, Ioana Karla Comariţa, Alexandra Vîlcu, Anastasia Procopciuc, Gabriela Tanko, Adriana Georgescu

**Affiliations:** Pathophysiology and Pharmacology, Institute of Cellular Biology and Pathology, ‘Nicolae Simionescu’ of the Romanian Academy, Bucharest, Romania

**Keywords:** microRNAs, platelets, microvesicles, atherosclerosis, animal model

## Abstract

**Aim:** The aim of this study was to analyze the expressed profiles of miRNAs in plasma, platelets, and platelet-derived microvesicles (PMVs) obtained from experimental induced atherosclerosis animal model and to investigate the effect of EPC transplantation on these profiles.

**Methods:** Seventeen selected circulating miRNAs (miR-19a,-21,-126,-146a,-223,-26b,-92a,-222,-210,-221,-143,-10a,-145,-155,-34a,-204, and miR-214) were individually analyzed in plasma, platelets, and PMVs isolated from peripheral blood of hypertensive-hyperlipidemic hamsters treated or not with endothelial progenitor cells (EPCs), and of healthy hamsters taken as control group.

**Results:** Comparative with control group, in hypertension associated with hyperlipidemia the investigated miRNA expression profiles were different: (i) in plasma, the levels of all investigated miRNAs were significantly increased, the highest enhances being noticed for miR-21,-146a,-221,-143,-34a, and miR-204; (ii) in platelets, the expressions of almost all miRNAs were significantly elevated, remarkable for miR-126,-146a,-223,-222, and miR-214, while levels of miR-143, miR-10a, and miR-145 were significantly reduced; (iii) in PMVs, numerous miRNAs were found to have significantly increased levels, especially miR-222,-221,-210, and miR-34a, whereas expressions of various miRNAs as miR-223,-214,-146a,-143,-10a, and miR-145 were significantly decreased. The treatment with EPCs had the following reverse effects: (i) in plasma, significantly reduced the expression of miR-26b,-143,-34a,-204, and miR-214; (ii) in platelets, significantly decreased the levels of almost investigated miRNAs, with remarkably diminishing for miR-126 and miR-221; and (iii) in PMVs, significantly lowered the expression of some miRNAs, with considerably reductions for miR-222,-221,-210, and miR-19a, while the level of miR-214 was found elevated.

**Conclusions:** The present study revealed that miRNAs have differential expression profiles in plasma, platelets, and PMVs under hypertension associated with hyperlipidemia conditions. The different miRNA profile in PMVs compared with platelets indicated an active mechanism of selective packing of miRNAs into PMVs from maternal cells; various miRNAs such as miR-19a,-21,-126,-26b,-92a,-155,-204,-210,-221,-222, and−34a delivered by PMVs may contribute to enrichment of circulating plasma miRNA expression. In addition, our study showed that the EPC-based therapy can regulate the expressions of investigated miRNAs into the three sources. These results provide novel information that could help in finding potential targets for the development of new therapeutic strategies in the cardiovascular disease.

## Introduction

Atherosclerosis is a chronic immune-inflammatory disorder that integrates multiple cell types and a diverse set of inflammatory mediators (1). Several experimental data has revealed an essential role for microRNAs (miRNAs) in regulation of the cellular and molecular processes related to atherosclerosis development, extending from risk factors, to plaque initiation and progression, up to atherosclerotic plaque rupture (2).

The discovery of miRNAs and their role in the regulation of gene expression in humans is one of the most exciting scientific discoveries in the last years. The miRNAs are highly conserved, single-stranded non-coding RNA molecules, with 22 nt in length, that exert post-transcriptional effects on gene expression by promoting the degradation of mRNA target and/or inhibiting mRNA translation. Gene regulation modulated by miRNAs is involved in almost biological processes in mammals and has important roles in health and disease states (1). Moreover, numerous miRNAs have been found outside of the cells, and the levels of specific miRNAs in circulation have great potential as biomarkers for different pathological conditions, such as atherosclerosis (3). Thus, expressions of circulating miRNAs have been described to be associated with cardiovascular disease (CVD): miRNA-19, miRNA-21, miRNA-126, miRNA-146, and miRNA-223 (4); with coronary artery disease (CAD): miRNA-92a, miRNA-221, and miRNA-222 (5) and with hypertension, miR-143 (5). Furthermore, miR-155 and miR-145 have been considered as diagnostic biomarkers of the CAD (6). Additionally, due to its involvement to the pathogenesis of various human disorders, miR-214 has been regarded as a promising marker in the prognosis, diagnosis and treatment of CVD (7).

Mostly miRNAs in the circulation have been shown associated with microparticles, exosomes, lipoproteins, or protein complexes (8, 9). Microvesicles (MVs), also known as microparticles (MPs), are small vesicles of 100 to 1,000 nm in size, released by cells in response to activation or apoptosis. A growing data suggested that platelet-derived microvesicles (PMVs), the most abundant MVs in the circulation, are key regulators of inflammation, hemostasis, and angiogenesis (10, 11). Moreover, it has been reported that the circulatory PMV levels are increased during several disease such as coagulation disorders, CVD, diabetes mellitus, rheumatoid arthritis, cancers, and infections, indicating their potential contribution to disease and their development as a biomarker (4, 10, 11). In patients with atherosclerosis, PMV number increase was observed to correlate with numerous parameters including carotid artery intima-media thickness, lipid-rich atherosclerotic plaques, and plaque burden (11). The PMVs are involved in all stages of atherogenesis: (i) initiate endothelial apoptosis via the insulin-like growth factor-1 receptor, by miR-223 transfer to endothelial cells (ECs) (12); (i) increase endothelial permeability inducing dysfunction of vascular endothelium, the initial step in the development of atherosclerosis (13); (iii) interact with leukocytes by binding of P-selectin to P-selectin glycoprotein ligand-1, promoting the accumulation of leukocytes at the site of endothelial injury and increasing the infiltration of leukocytes into the intima (14); (iv) transfer the pro-atherogenic cytokine RANTES to ECs and induce monocytes and ECs to release pro-inflammatory cytokines such as interleukins (IL): IL-8, IL-1β, IL-6, and TNF-α, further increasing the adhesion and the infiltration of leukocytes (15, 16); (v) induce macrophage apoptosis by encapsulating active caspase-3 (17); (vi) stimulate smooth muscle cells (SMCs), leading to the SMC migration from the media to the intima, thus increasing lesion progression (18). In a previous study we revealed that administration of atherosclerotic origin PMVs significantly increased the levels of pro-inflammatory cytokines and chemokines, the lipid deposits in the liver and the vascular wall, promoted the macrophage infiltration in liver and atherosclerotic lesions in thoracic aorta and in resistance arteries, augmented arterial resistance and dysfunction, and aggravated the atherosclerosis process (19).

In the stages of atherosclerotic lesion development, augmented hemodynamic shear stress due to luminal stenosis associated with plaque, as well as the accumulation of oxidized low-density lipoprotein (LDL), can activate platelets, that further can interact with ECs of inflamed arteries and stimulate generation of pro-inflammatory PMVs (11). Although no longer able to generate RNA *de novo*, platelets contain mRNA, YRNA fragments and premature miRNAs that were inherited from megakaryocytes. Some miRNAs, such as miR-126 and miR-223, have an important role in regulating platelet reactivity. In this sense, it has been demonstrated that miR-223 is involved in aggregation and thrombus formation, targeting the P2Y12, a purinergic receptor known to intensify aggregation induced by all known platelet agonists (20, 21). Moreover, platelet miR-223 can regulate vascular EC apoptosis, while its expression can be enhanced by stimulation with thrombopoietin (22). It has been shown that miRNA-126 has an essential role in platelet activation, since single-nucleotide polymorphism that accelerates miRNA-126 processing is correlated with level increase of platelet activation markers in plasma, and administration of antagomiRs to miRNA-126 diminishes platelet activation in mice (23). Furthermore, levels of miR-126, that indicate platelet activation, are regarded as a prognostic marker for cardiovascular event. Both, miR-126 and miR-223 have been profiled, as biomarkers, for identification of the risk of myocardial infarction (22). It was suggested that in patients with acute coronary syndromes, miR-223 profiling might help in the evaluation of response to antiplatelet agents (22). Regarding miR-126, it was showed that *in vivo* aspirin administration induced platelet inhibition and can reduced the circulating levels of miR-126 derived from platelets (24).

Endothelial progenitor cells (EPCs) have generated a significant attention as potential novel diagnostic/prognostic biomarkers for vascular integrity and also for use in therapeutic clinical approaches (25). Furthermore, the contribution of EPCs to the restoration of endothelial monolayer induced the possibility to use EPCs as a novel preventative and/or treatment strategy for atherosclerosis (26). In a previous study it was shown that the EPC-based therapy suppresses the development of atherosclerosis, reduces hepatic lipid and macrophage accumulation with the consequent alleviation of dyslipidaemia and hypertension in a hypertensive-hyperlipidemic hamster model (19).

The research in platelets and PMV field has grown in recent years, and in the near future due to the technologies arrive at the horizon will probable advance and will take a new position in therapy of cardiovascular disease. Consequently, the aim of this study was to analyze the differentially expressed profiles of miRNAs in plasma, platelets, and PMVs obtained from experimental induced atherosclerosis animal model and to investigate the effect of EPC transplantation on these profiles.

## Results

As our purpose was to investigate the miRNA profiles with key role in the CVD development in plasma, platelets, and PMVs obtained in peripheral blood collected from hypertensive-hyperlipidemic hamster (HH group; which could mimic human atherosclerosis), we individually quantified several miRNAs employing hybridization probes (miRNA TaqMan assays) in each investigated sample. Furthermore, we explored the changes induced by EPC transplantation on profiles of these miRNAs in plasma, platelets, and PMVs from HH group injected with EPCs (HH-EPCs group). The experimental animal models were characterized previously by our group in different papers, illustrating the plasma profile for glucose, cholesterol, triglyceride concentrations, and also the blood pressure and heart rate (27–31). Moreover, our data revealed that hypertension associated with hyperlipidemia is accompanied by structural modifications, substantial accumulation of collagen, lipid and macrophages in atherosclerotic lesions, expression of pro-inflammatory molecules by the vessel wall, the alteration of vascular tone, enhanced release of MVs and reduced EPCs (30). The EPC transplantation re-established plasmatic parameters (cholesterol and triglyceride concentrations), blood pressure, heart rate, cytokine and chemokine profiles, PMV pro-thrombotic activity, and EPC paracrine activity reflected by cytokine/chemokine detection (19).

### Circulating miRNA Levels Are Changed by Atherosclerotic Milieu

First of all, the 17 selected circulating miRNAs (miR-19a, miR-21, miR-126, miR-146a, miR-223, miR-26b, miR-92a, miR-222, miR-210, miR-221, miR-143, miR-10a, miR-145, miR-155, miR-34a, miR-204, and miR-214) were individually quantified in plasma obtained from each animal in HH and HH-EPCs groups and compared to those in plasma collected from healthy hamsters taken as control group (C group).

**Figure 1:**
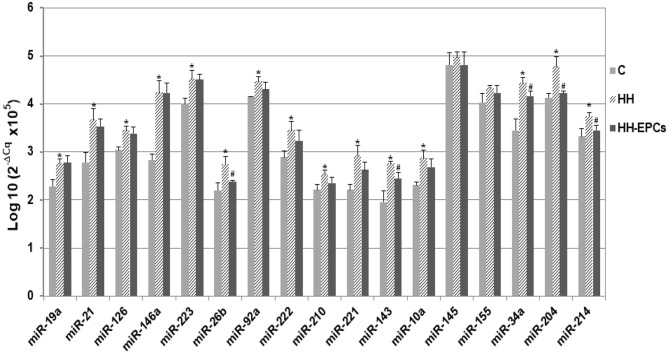
The quantification of 17 circulating miRNA expressions in plasma obtained from hamster groups: control (C), hypertensive-hyperlipidemic (HH), and HH treated with EPCs (HH-EPCs). Data were expressed as log-transformed values of individual 2^−ΔCq^ values (Δ*Cq* = Cq has-miRNA-Cq cel-miR-39), multiplied to obtain a positive values distribution; The statistically significant differences between the groups were calculated, and represented as *p≤ 0.05 for values vs. C group, and as ^#^*p* ≤ 0.05 for values vs. HH group (one way ANOVA analysis).

Results showed that almost all selected miRNAs had levels significantly increased in the plasma of HH hamsters compared to those quantified in plasma collected from hamsters in C group (^*^p ≤ 0.05, Figure 1). The highest enhances were acquired for miR-21, miR-146a, miR-221, miR-143, miR-34a, and miR-204 (about 1.3 times up to 1.5 times), while expressions of miR-145 and miR-155 were slightly increased in HH group. It is interesting to note that, after EPC therapy, although for all investigated circulating miRNAs the expressions decreased, only for some of them, namely miR-26b, miR-143, miR-34a, miR-204, and miR-214, the reductions were significantly (^#^*p* ≤ 0.05, Figure 1).

### The miRNAs Packaged Into PMVs Are Different From Those in the Platelets in Atherosclerosis

Next we analyzed the same 17 miRNAs in platelets and PMVs isolated from C, HH, and HH-EPCs groups.

**Figure 2:**
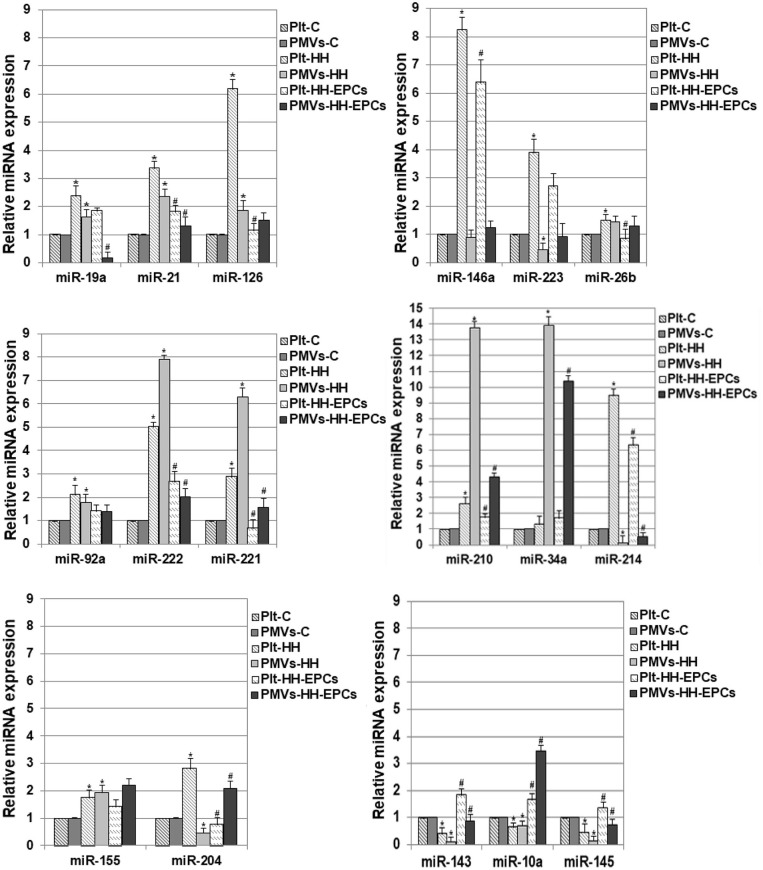
Relative expression levels of 17 differentially expressed miRNAs in platelets (Plt) and platelet-derivate microvesicles (PMVs) obtained from hamster groups: control (Plt-C and PMVs-C), hypertensive-hyperlipidemic (Plt-HH and PMVs-HH), HH treated with EPCs (Plt-HH-EPCs and PMVs-HH-EPCs). The statistically significant differences between the groups were calculated, and represented as *p ≤ 0.05 for values vs. C group, and as ^#^*p* ≤ 0.05 for values vs. HH group (one way ANOVA analysis).

The quantitative reverse transcription polymerase chain reaction analysis of miR-19a, miR-21, miR-126, miR-146a, miR-223, miR-26b, miR-92a, miR-222, miR-221, miR-210, and miR-214 revealed that the levels of these miRNAs were significantly increased in platelets from HH group compared to platelets isolated from C group, and were lower in platelets isolated from HH-EPCs group compared to the values took for HH group (^*^p ≤ 0.05; Figure 2). The highest increases in HH group were noticed for miR-126, miR-146a, miR-223, miR-222, and-214 and they were by ~6.19-, ~8.25-, ~3.89-, ~5.02-, and by ~9.48-fold, respectively, suggesting that expressions of these miRNAs are relevant for activated platelets in atherosclerosis (Figure 2). The EPC transplantation remarkably reduced expressions of the miR-126 and miR-221 in platelets by ~5.42- and ~4.36-fold (Figure 2) recommending these miRNAs as suggestive players for EPC effect on platelets in hypertension associated with hyperlipidemia. Interesting, for miR-34a expression in platelets we did not find any significant differences between the values obtained from hamsters in all the three experimental groups (Figure 2).

In PMVs, we found significantly increased levels for miR-19a, miR-21, miR-126, miR-92a, miR-222, miR-221, miR-210, and miR-34a in HH group compared to values obtained from hamsters in C group, while transplantation of the EPCs reduced expression of these miRNAs, compared to values obtained at HH group (^*^p ≤ 0.05; Figure 2). The highest enhance in PMVs from HH group was obtained for miR-222, miR-221, miR-210, and miR-34a the rising being by ~7.89-, ~6.29-, ~13.76-, and ~13.89-fold, respectively (Figure 2). In PMVs from HH-EPCs group, the important decreases were found for miR-222, miR-221, miR-210 and miR-19a expressions (by ~3.93-, ~4-, ~3.18-, and ~ 9.85-fold, respectively) compared to values obtained for PMVs in HH group. A slightly enhance was found for miR-26b expression, while the quantified levels for miR-223, miR-214, and miR-146a were decreased in PMVs isolated from HH group compared to C group (^*^p ≤ 0.05; Figure 2). A considerably increase of ~4.58-fold it was obtained for miR-214 expression in PMVs from HH-EPCs group compared to HH group (Figure 2), indicating that EPC transplantation can regulate the miR-214 expression from PMVs in hypertension associated with hyperlipidemia as well.

Others important miRNAs like miR-155, miR-204, miR-143, miR-10a, and miR-145 have been analyzed by qRT-PCR in our study (Figure 2).

Expressions of miR-155 and miR-204 were significantly increased in platelets from HH group, comparative with C group (^*^p ≤ 0.05; Figure 2), while EPC transplantation induced reduction of these miRNA expressions, especially for miR-204, for which the decrease was about 3.6 times compared to HH group (^*^p ≤ 0.05; Figure 2). For PMVs, our results showed that the miR-155 expression was increased in HH group, while the miR-204 expression was diminished compared to values obtained for PMVs in C group. In HH-EPCs group, an increase of expression for both investigated miRNAs was found when we compared to values of miRNA expressions from HH group, especially for miR-204 that increased by ~4.6-fold (^*^p ≤ 0.05; Figure 2).

The analysis of miR-143, miR-10a, and miR-145 expressions in platelets and PMVs obtained from HH group disclosed that these were significantly reduced compared to the same miR expressions in platelets and PMVs from C group (^*^p ≤ 0.05; Figure 2), while in platelets and PMVs from HH-EPCs group the miR-143, miR-10a, and miR-145 expressions were elevated vs. HH group (^*^p ≤ 0.05; Figure 2). To point out that, transplantation of EPCs induced a markedly increase of miR-143 in platelets (by ~4.5-fold), and in PMVs of miR-143, miR-10a, miR-145 expressions (by ~9.18-, ~ 4.99-, and ~5.55-fold, respectively) (Figure 2).

Noticeable, the levels of investigated miRNAs were decreased in PMVs from HH group compared to values, with the exception of miR-222, miR-221, miR-210, and miR-34a, which surprisingly had a pronounced augmentation by ~1.57-, ~2.17-, ~5.18-, and by ~10.51-fold, respectively (^*^p ≤ 0.05; Figure 2). Also, we observed that levels of miR-223, miR-146a, miR-214, and miR-204 were reduced in PMVs from HH group comparative with values obtained in C group, while in platelets from HH group these miRNAs had a great increase (Figure 2).

The obtained data have revealed that, in experimental induced atherosclerosis the profile of investigated miRNAs in plasma is different from that of platelets and MPVs. Thus, in plasma, the significantly enhances were for miR-21, miR-146a, miR-221, miR-143, miR-34a, and miR-204. In platelets and PMVs, only a few miRNAs were found to have the same behavior: miR-222 expression was increased, while miR-143, miR-10a, and miR-145 expressions were reduced. Interesting, while in platelets we detected a notable increase of miR-223, miR-146a, miR-214 expressions, in PMVs the same miRNAs have reduced levels, suggesting that probably these miRNAs could be incorporated into exosomes rather than in microvesicles.

Moreover, our findings disclosed that transplantation of EPCs improved the levels of the 17 miRs affected by hypertension associated with hyperlipidemia, especially by reducing expression of miR-26b, miR-143, miR-34a, miR-204, and miR-214 in plasma. Also, the EPC therapy improved the levels of these miRNAs in platelets and PMVs, having a crucial influence mostly on miR-126, miR-221 in platelets, and on miR-222, miR-221, miR-210, miR-19a, and miR-214 in PMVs, indicating that the possible positive effects of EPCs are exercised through the molecules involved in signaling pathways regulated by these miRNAs.

## Discussion

Over the last years, numerous groups have revealed the potential role of circulating miRNAs as biomarkers for CVD (9, 32). The miRNA content in the circulation may reflect the activation state of circulating cells and may provide information for cell activation and tissue injury in response to cardiovascular risk factors and diseases (9). Moreover, the possibility to investigate miRNAs in the circulation has increased due to their remarkably stability, even under conditions as boiling, low or high pH, long-time storage at room temperature, and multiple freeze-thaw cycles (33).

Data from the literature suggest that platelets are responsible for up to 50% of the extracellular vesicles (microvesicles and exosomes) in the blood (34). Consequently, the microRNA content of the plasma, which includes all extracellular vesicles from the blood, is profoundly influenced by the microRNA content of platelet-derived extracellular MVs. Furthermore, due to their various benefits, including capacity to be released locally, the ease way to travel through the body, the ease way to obtain them, and their low immunogenicity, MVs are considered as novel and specific pharmacological targets to manage atherothrombosis and, also as potential therapeutic tools for the administration of drugs and other molecules, including miRNAs (35). Additionally, it was revealed that PMVs act as essential functional effectors that link inflammation, hypercoagulability and neovascularization contributing to the enhancement of atherosclerotic lesion development and to thrombosis (36).

In view of these findings, we selected a subset of 17 miRNAs related to CVD (miR-19a, miR-21, miR-126, miR-146a, miR-223, miR-26b, miR-92a, miR-222, miR-210, miR-10a, miR-221, miR-143, miR-10a, miR-145, miR-155, miR-34a, miR-204, and miR-214) and we analyzed their distribution in plasma, platelets, and PMVs isolated from atherosclerotic animal model compared to control group, and also we investigated the effect of EPCs treatment on these profiles.

In our study, we showed that in plasma from hypertensive-hyperlipidemic hamsters, all investigated miRNAs were augmented compared to values obtained in healthy animals, and the highest levels were noticed for miR-21, miR-146a, miR-221, miR-143, miR-34a, miR-204. These results are in agreement with several studies that indicated significant increases in levels of: (i) miR-21 and miR-210 in serum samples from patients with peripheral arterial disease, specifically atherosclerosis obliterans (37); (ii) miR-34a and miR-146 in serum obtained from patients with newly diagnosed type II diabetes (38); (iii) miR-221 and miR-222, that indirectly repress endothelial nitric oxide synthase required for EPC functions, and (iv) miR-92a in patients with CAD (39). The levels of miR-92a, that have a pivotal role in regulating EC response to shear stress, increased also in atherosclerosis (40). Moreover, it has been demonstrated that circulating miR-19a expression was higher in the patients with acute myocardial infarction, the most serious complication of CAD (41). Several clinical and animal studies revealed that miR-143 and miR-145 contributed to pathogenesis of atherosclerosis, by their involvement in regulation of ECs, vascular SMCs, and circulating blood cells functions (42). It has been shown that miR-223 levels were enhanced in ApoE- and LDL-receptor deficient mice under high-fat diet and also, in patients with familiar hypercholesterolemia, and might be involved in regulating of liver gene expression due its role as intercellular communication system (8). Furthermore, the higher miR-223 levels consistently anticipated future cardiovascular deaths of the patients with symptomatic CAD, suggesting that miR-223 could also be used as prognostic marker for the risk of cardiovascular death (43). Interesting, in a study performed on children at age 10 to 12 years, with increased risk of metabolic syndrome, and augmented miR-126 plasma levels, it was suggested that miR-126 could actively participate in the development of insulin resistance and could represent an early marker of metabolic dysfunction (44).

In order to explore whether circulating plasma miRNAs reported as biomarkers for CVD could be associated with miRNA carried by microvesicles released from activated platelets we evaluated the same miRNA profile in plasma and also in PMVs and platelets, their maternal cells. Our findings showed that PMVs isolated from hypertensive-hyperlipidemic hamsters contain increased levels of miR-19a,-21,-126,-26b,-92a,-155,-204, and a remarkably augmentation for miR-222,-221,-210, and-34a. The same miRNAs have been found elevated also in platelets from hypertensive-hyperlipidemic hamsters. Notable, rise of miR-126 expression was much higher in platelets than in PMVs. Thus, PMVs by their miRNA content may contribute to the enhance of circulating miRNA expression in plasma of experimental induced atherosclerosis model, mainly of miR-19a,-21,-126,-26b,-92a,-155,-204,-210,-221,-222, and-34a levels. Our results are supported by other studies from literature. Therefore, it has been shown that levels for miR-92a, miR-21, and miR-126 in MVs were augmented in patients with CAD, suggesting that MVs may contribute to the regulation of circulating miRNA expression (45). Moreover, miR-126 and miR-21 were found at high levels in platelets and PMVs at patients with CVD (46), and the elevated amounts of these miRNAs in PMVs were correlated with their circulating levels (47). It has been shown that miRNA-19, involved in cardiac hypertrophy and angiogenesis, was significantly up-regulated in PMVs (4).

Moreover, expressions of miR-143,-10a, and-145 were reduced both in PMVs and platelets comparative with sample from control hamsters suggesting that perhaps PMVs do not have an influence on these miRNA levels in plasma of hypertensive-hyperlipidemic animals. Interestingly, although the expressions of miRNA-146a,-223, and-214 were significantly greater in platelets, in PMVs were decreased. The differences in the miRNA profile in platelets and PMVs indicate that platelets may have a selective mechanism to packing miRNAs into PMVs. Furthermore, there results indicated that at high levels of circulating miRNA-146a,-223, and-214 in hypertension associated with hyperlipidemia conditions may contribute other circulating cells, but not platelets. In literature, was reported that miR-223, as well miR-126, are highly expressed in platelets in patients with CVD (46) and are considered biomarkers associated with the risk of myocardial infarction (48).

In our previous study we demonstrated that transplantation of EPCs in the experimental induced atherosclerosis animal model reduced platelet activation, modulated their pro-inflammatory and antithrombogenic properties (28) and had a beneficial impact on EPCs-platelets relationship, improving their functions (49). Thus, in the current study we followed the EPC treatment effects on the profile of the same 17 miRNAs in plasma, platelets and PMVs. Our findings reveal that EPC transplantation in hypertensive-hyperlipidemic hamsters improved the levels of the 17 plasma investigated miRNAs, especially by reducing expression of miR-26b, miR-143, miR-34a, miR-204, and miR-214. In platelets and PMVs, EPC treatment diminished the levels of almost all of investigated miRNAs, having a crucial influence in particular on miR-126, miR-221 in platelets, and on miR-222,-221,-210,-19a in PMVs, indicating that these miRNAs could mediate the beneficial effects of EPC, observed in our previous study. Expression of miR-214 was also reduced, in both plasma and platelets, after EPC transplantation, compared with values obtained at atherosclerotic animals (HH group). Our data indicating the beneficial effects of EPC treatment on miRNA profile of platelets and PMVs in hypertension associated with hyperlipidemia, could explain the improvement of platelet functions after EPC treatment at HH group, observed in the previous studies.

In conclusion, our findings bring new information concerning PMV involvement in the regulation of the miRNA profile in plasma, in hypertension associated with hyperlipidemia. Numerous miRNAs, such as miR-19a,-21,-126,-26b,-92a,-155,-204,-210,-221,-222, and-34a delivered by PMVs may contribute to augmentation of circulating plasma miRNA expression. Additionally, our present data have shown that the EPC-based therapy modified the expressions of these miRNAs in the three compartments. The miRNAs contained by PMVs can be internalized by the different recipient cells involved in atherosclerosis development, e.g., macrophages, ECs, leukocytes, vascular SMCs, and hepotacytes, modulating the gene expression and regulating their functions. Although, further studies are needed to elucidate the exact role of specific platelet miRNAs in vascular biology and atherosclerosis progress and involved signaling pathways, our data bring answers which may help to develop the forthcoming therapeutic applications for cardiovascular disease.

## Materials and Methods

### Hamster Models of Atherosclerosis and Transplant

The experiments were carried out on plasma, platelets and PMVs, obtained from the blood of Golden Syrian hamsters (3 months old, *n* = 30) divided in three equal groups: (1) C (control hamsters), age-matched normal healthy animals which received an ordinary hamster diet; (2) HH (simultaneously hypertensive-hyperlipidemic hamsters), fed with ordinary diet enriched with 3% cholesterol, 15% butter and 8% NaCl, for 4 months; (3) HH-EPCs (HH hamsters treated with EPCs), animals that received 1 x 10^5^ EPCs (isolated from C group) in a volume of 300 μl, in a dose per month, via the retro-orbital venous sinus injection, during 4 months of diet specific to the HH hamster, to prevent atherosclerosis process; (27–29, 49, 50) and (19, 30, 31). At the end of the experiment, the hamsters were sacrificed for biochemical assays.

The use of animals in experiments and the procedures necessary to carry out the research study have approved by the Ethics Committees from Institute of Cellular Biology and Pathology “Nicolae Simionescu,” and they have been realized in agreement with National, European and International legislation on the use of experimental animals in biomedical research (29, 50).

### EPC Isolation and Sorting

Peripheral blood mononuclear cells were obtained by density-gradient centrifugation using Histopaque-1077 as described by Georgescu et al. (19, 30). Briefly, opaque interface containing the mononuclear cells (MNCs) was aspirate and transfer into clean tube, after centrifugation at 400 x g for 30 min, of 1 ml whole blood (collected from the retro-orbital venous sinus, ~1.5 ml per hamster) onto 3 ml Histopaque-1077. Next, the opaque interface was mixt with 10 ml isotonic PBS solution containing 2% fetal bovine serum (FBS) and centrifuged at 256 x g for 10 min. The supernatant was aspirated and discarded, and after three times repetitive washings the MNCs pellet was resuspended in 10 ml PBS.

The EPCs from C hamsters were sorted in PBS, from MNCs, at the same number, 1 x 10^5^/sample, applying the flow cytometry technique (MoFlo flow cytometer equipped with high-speed cell sorter, Dako, USA), using the specific antibodies for VEGF-R2 (KDR or Flk-1) and CD34. The CD34 and VEGF-R2 are markers for late or mature EPCs, that are found more than early EPCs (CD133^+^, CD34^+^, VEGF-R2^+^), in the peripheral circulation of adults (30, 51). Importantly, the monthly blood collection from C hamsters via the retro-orbital venous sinus, to obtain EPCs, did not affect the health status of animals.

### Preparation of Platelet-Free Plasma (PFP), the Source for Circulating PMVs

Plasma PMVs were separated according to the method reported by Georgescu et al. (30, 52). Briefly, the venous blood collected from retro-orbital venous sinus (~1.5 ml per hamster), in 0.138 M tri–sodium citrate 9/1 (vol/vol) was centrifuged at 1,000 x g for 15 min, at 15°C to obtain platelet rich plasma (PRP). PFP obtained after centrifugation of PRP at 2,500 x g for 15 min, at 15°C, was centrifuged again at 13,000 x g for 5 min, at 15°C allowing the collection of PMVs in the supernatant.

### PMV Sorting

PMVs were sorted from PFP isolated from each hamster, according to Georgescu et al. (30, 52), using the MoFlo flow cytometer equipped with high-speed cell sorter (Dako, USA) and specific antibodies, annexin V conjugated with fluorescein isothiocyanate (FITC) (for PS) and integrin αIIb (M 148) conjugated with phycoerythrin (PE) (for CD41).

### Platelet Isolation

Platelets were separated according to the method reported by Alexandru et al. (27, 28, 50). Briefly, the hamsters were slightly anesthetized with 2% isoflurane in oxygen (2.4 l/min), and venous blood was collected from the retro-orbital venous sinus (~1.5 ml per hamster), in ACD buffer (2.73% citric acid, 4.48% trisodium citrate, and 2% glucose) and centrifuged at 400 x g for 10 min to obtain the PRP. Next, platelets were isolated by centrifugation of PRP at 600 x g for 10 min, and were suspended in calcium-free Hepes buffer (pH 7.0) supplemented with 1% BSA and 0.15 U/ml apyrase. Analysis of the suspension by phase contrast microscopy showed that this was devoid of erythrocytes and leukocytes and the platelets were not aggregated.

### The MicroRNA Detection by Real-Time Quantitative-Polymerase Chain Reaction (RT q-PCR) assay

The miRNA expressions in platelet-poor plasma (but rich in total MVs, including PMVs), platelets and PMVs isolated from peripheral blood of C, HH, and HH-EPCs hamsters were measured by miRNA TaqMan assay.

First of all, total RNA from plasma was obtained using miRNeasy Serum/Plasma Kit (Qiagen, Dusseldorf, Germany), while total RNA of platelets and PMVs was isolated using miRNeasy Micro Kit (Qiagen, Dusseldorf, Germany) according to the manufacturer's guidelines. In order to isolate plasma circulating miRNAs, the blood samples were centrifuged at 1,900 x g (3,000 rpm), 10 min at 4°C to remove blood cells and platelets from plasma, and the resulting supernatant was centrifuged at 16,000 x g, 10 min at 4°C to remove additional cellular nucleic acids attached to cell debris. The latter centrifugation step significantly reduced the amount of cellular or genomic DNA and RNA in samples. At the end of the procedure, the RNAs were eluted from the silica columns with 15 μl of RNase-free water, and after concentration measurement using the NanoDrop 2000c spectrophotometer, were kept at −80°C until examinations. In the next step, the reverse-transcription was achieved using TaqMan MicroRNA ReverseTranscription Kit in combination with TaqMan-Gene Expression Master Mix and miRNA specific stem-loop primers of miRNA TaqMan Assays on a Veriti real-time PCR system (Applied Biosystems, Life Technologies Foster City, CA, USA). The miRNA specific stem-loop primers used were hsa-miR-19a-3p (ID:000395), hsa-miR-21-5p (ID: 000397), hsa-miR-126-3p (ID:002228), hsa-miR-146a-5p (ID:000468), hsa-miR-223-3p (ID: 002295), hsa-miR- 26b-5p (ID: 000407), hsa-miR-92a-3p (ID:000431), hsa-miR-222-3p (ID: 002276), hsa-miR-221-3p (ID:000524), hsa-miR-143-3p (ID:002249), hsa-miR-10a-5p (ID:000387), hsa-miR-145-5p (ID:002278), hsa-miR-155-5p (ID: 002623), hsa-miR-204-5p (ID:000508), hsa-miR-210-3p (ID: 000512), hsa-miR-34a-5p (ID:000426), hsa-miR-214-3p (ID: 002306).

The VIIA7 Software v1.2 (Applied Biosystems) with the automatic quantification cycle (Cq) setting was used to analyze the data. MiRNAs levels in plasma isolated from HH and HH-EPC hamsters were expressed relative to those obtained in C plasma with normalization to an endogenous control (cel-miR-39, 000200). The miRNAs with Cq values over 35 were omitted from further analysis. Fold change values of circulating miRNAs analyzed in the pooled plasma of HH and HH-EPC hamsters were expressed relatively to plasma C group. Fold change (2^−ΔΔCq^) values represent the normalized miRNA expression (2^−ΔCq^) in the test sample divided to the normalized miRNA expression (2^−ΔCq^) in the C sample. Normalized expression of a specific human miRNA is given relative to that of exogenously added cel-miR-39 (ΔCq = Cq hsa-miRNA–Cq cel-miR-39). In order to obtain a distribution of positive values, data were expressed as log-transformed values of multiplied individual 2^−ΔCq^ values.

The relative expression level of each miRNA isolated from platelets and PMVs, respectively, was normalized to U6 snRNA (ID:001973) and quantified as 2^−ΔΔCq^ (ΔCq = Cq hsa-miRNA - Cq U6) (53). The Cq is the fractional cycle number at which the fluorescence of each sample passes a fixed threshold. The fold change was generated using the equation 2^−ΔΔCq^. Fold change values greater than 2 indicate a positive or up-regulation and fold change values less than 1 indicate a negative or down-regulation.

## Data Analysis

For the quantification and comparison of the data, One-Way ANOVA method and Bartlett's test in GraphPad Prism 7.02 programme were applied. The statistically significant differences between the groups were calculated, and represented as *P* ≤ 0.05 values.

## Data Availability Statement

All datasets generated for this study are included in the article/supplementary material.

## Ethics Statement

The animal study was reviewed and approved by Ethics Committees from Institute of Cellular Biology and Pathology Nicolae Simionescu.

## Author Contributions

NA, AC, and AG contributed to conception and design. NA, AC, MN, IC, AV, and AP performed the experiments. NA, AC, MN, GT, and AG contributed to data analysis, and interpretation. NA, AC, and AG wrote the paper. AG provided a critical revision of the manuscript for important intellectual content. NA, AC, MN, IC, AV, AP, GT, and AG agrees to be accountable for all aspects of work ensuring integrity and accuracy.

### Conflict of Interest

The authors declare that the research was conducted in the absence of any commercial or financial relationships that could be construed as a potential conflict of interest.
